# The Dynamic Nature of Caregiving in Advanced Heart Failure With Left Ventricular Assist Device Support: A Systematic Review and Thematic Synthesis

**DOI:** 10.1007/s11897-026-00758-9

**Published:** 2026-04-30

**Authors:** Magda Eriksson-Liebon, Naoko P. Kato, Binyamin Ben Avraham, Tuvia Ben Gal, Tiny Jaarsma, Anna Strömberg, Maria Liljeroos

**Affiliations:** 1 Department of Health, Medicine and Caring Sciences (HMV), Linköping University, Kungsgatan 40, Norrköping, S-601 74 Sweden; 2 Heart Failure Unit, Department of Cardiology, Rabin Medical Centre, Petah Tikva, Israel; 3 Grey school of Medicine, Tel-Aviv University, Tel-Aviv, Israel; 4 Department of Cardiology, University Medical Center Utrecht, Utrecht, The Netherlands; 5 Department of Cardiology, Linköping University hospital, Linköping, Sweden; 6 Centre for Clinical Research Sormland, Uppsala University, Eskilstuna, Sweden

**Keywords:** Heart failure, LVAD, Caregivers, Experiences, Systematic review, Thematic synthesis

## Abstract

**Purpose of Review:**

Advanced heart failure is a growing global health challenge, and the use of Left Ventricular Assist Devices (LVADs) as a long-term treatment has increased. This development shifts substantial responsibility to informal caregivers, who play a crucial role in patients’ daily management and wellbeing. However, knowledge about caregivers’ experiences across the entire LVAD trajectory remains limited. This review aims to synthesize available evidence on informal caregivers’ experiences of everyday life when supporting an adult patient with advanced heart failure treated with an LVAD.

**Recent Findings:**

This systematic review and thematic synthesis included 17 studies. The analysis identified ten descriptive themes related to caregivers’ experiences, which were further synthesized into four analytical themes: Before LVAD implantation, Early post-LVAD, Later post-LVAD, and End of the LVAD journey. These themes illustrate the evolving and dynamic nature of caregiving across the LVAD trajectory. In addition, one overarching analytical theme, The caregiver’s network of support, was identified.

**Summary:**

The findings highlight caregivers’ experiences of both receiving and providing support through formal and informal networks throughout the LVAD trajectory, emerging as a continuous and integral component of caregiving. The review underscores the importance of sustained and structured support for caregivers across the entire LVAD pathway, rather than support limited to specific time points.

**Supplementary Information:**

The online version contains supplementary material available at 10.1007/s11897-026-00758-9.

## Introduction

Advanced heart failure (HF) is characterized by severe symptoms despite optimal medical therapy [1]. For these patients, left ventricular assist devices (LVADs) are a vital therapeutic option, either as a bridge to transplantability and transplantation (BTT), as a bridge to recovery (BTR) or as destination therapy (DT) for those ineligible for transplant [2]. Over the decades, LVAD technology has advanced considerably, shifting from pulsatile-flow to continuous-flow systems, which have demonstrated superior durability, reduced complications rate, and improved patient outcomes [3, 4]. Beyond prolonging survival, LVADs significantly improve functional capacity and quality of life for appropriately selected patients [4, 5].

However, the success of LVAD therapy depends not only on device innovations but also on the ability of informal caregivers to provide continuous support and assistance with daily management [6, 7].

An informal caregiver may be defined as “a person who provides unpaid care to someone with a chronic illness, disability or other long-lasting health or care needs, outside a professional or formal framework” [8]. These caregivers, often spouses, adult children, siblings, or close friends, assume extensive and demanding responsibilities in supporting patients throughout the course of LVAD support. Their tasks commonly include monitoring device function, managing power sources, performing sterile dressing changes, administering medications, and supporting mobility [9, 10]. Unfortunately, the role of the main caregivers in LVAD therapy is frequently underrecognized and thus overlooked [9, 11].

At a broader systems level, informal caregivers are indispensable: they provide the majority of long-term care and constitute a cornerstone of health and social care sustainability in the context of population ageing, increasing chronic illness, and rising demands on community-based care [12]. Clinical guidelines similarly emphasize the critical role of caregivers in LVAD therapy, in some cases making a designated caregiver a prerequisite for safe post-implantation management [13–15].

Caregiving in the context of LVAD therapy often begins even before implantation, as patients increasingly rely on physical, emotional, and practical support [16]. After implantation, these responsibilities intensify, and caregivers frequently experience substantial strain as they adapt to the complexities of the role [17]. Research consistently shows that caregiving has a profound impact on caregivers’ daily lives and well-being, with elevated stress, anxiety, and emotional exhaustion commonly reported [18]. Furthermore, the transition from traditional caregiving roles to specialized LVAD management may also disrupt family dynamics, adding to psychosocial strain [17]. Caregiving can also create financial strain, affect caregivers’ own health, and influence patient outcomes, as high caregiver distress is associated with poorer adherence and more frequent hospitalizations [19–21]. Yet, some caregivers report positive outcomes such as stronger emotional bonds and personal growth [22].

Although previous systematic reviews have described both burdens and rewards of caregiving, important gaps remain. Magid et al. [17] called for deeper understanding of caregivers’ experiences, particularly when patients have complex illness trajectories, while Streur et al. [18] highlighted limited longitudinal evidence across LVAD treatment pathways. Furthermore, qualitative findings remain fragmented, hindering an integrated understanding of caregiving throughout the LVAD trajectory. Therefore, a qualitative thematic synthesis was needed to integrate existing evidence and provide an understanding of caregivers lived experiences across the full LVAD trajectory, thereby informing the development of more targeted and effective support interventions.

### Aim

To synthesize available evidence on the informal caregivers’ experiences of everyday life when supporting an adult patient with advanced heart failure supported with an LVAD.

## Method

A qualitative systematic review with thematic synthesis was conducted to explore informal caregivers’ experiences of everyday life when supporting adults with advanced heart failure treated with an LVAD. This approach is appropriate for synthesising qualitative evidence to generate in-depth and nuanced understandings of caregivers’ lived experiences across different contexts [23].

### Design

This systematic review using thematic synthesis and in accordance to standard reporting guidelines, following the Enhancing Transparency in Reporting the Synthesis of Qualitative Research (ENTREQ) framework [24]. The review process followed the PRISMA (Preferred Reporting Items for Systematic Reviews and Meta-Analyses) guidelines [25]. The study protocol was registered in the PROSPERO International Prospective Register of Systematic Reviews (registration number CRD42025619587).

### Search Strategy

Six electronic databases were searched: PubMed, Embase, CINAHL, PsycInfo, Scopus and Cochrane Central. The search strategies were developed, peer reviewed, and executed by two medical librarians in accordance with the PRESS guideline [26]. A modified qualitative research filter and a modified systematic review filter in PubMed were applied. Limits included a publication date restriction to 2015 onwards, with conference publications excluded in Embase. All identified records were imported into EndNote 21, deduplicated using Tera-Tools, and the resulting dataset was re-imported into EndNote for screening. The final search was conducted on March 31, 2025, and an updated search was performed on November 13, 2025, using the same search strategy as the initial search. The updated search included studies published between March 31 and November 13, 2025. The complete search strategies are provided in the supplement file (see Supplement file 1).

### Eligibility Criteria

Studies were eligible for inclusion if they involved adult informal caregivers (family members, spouses, or other non-professional caregivers) providing care to adults with an LVAD in a home-based context, either as a bridge to transplantation or as destination therapy. All peer-reviewed qualitative studies were eligible for inclusion, regardless of methodological approach. Mixed-methods studies were included only if qualitative methodology and findings were clearly reported, and in such cases only the qualitative data were extracted. Eligible studies focused on caregiver experiences, perceptions, attitudes, beliefs, and the emotional, physical, or social factors influencing caregiving. The review was restricted to studies published in English within the last ten years (2015–2025). Exclusion criteria included studies on professional caregivers or healthcare professionals, purely quantitative research, non-peer-reviewed publications, and sources without full-text availability.

### Data Extraction

Data was extracted systematically by two independent reviewers (MEL and ML) using a predefined extraction table to ensure consistency and transparency. Extracted data included study identification (author(s), year of publication, country, and article title), study aim, design, participant characteristics (sample size and age), main topic and study results (categories, descriptive themes, and analytical subthemes). Discrepancies in data extraction were resolved through discussion and consensus among all authors. All studies were organized and managed using Rayyan, a web-based application designed for systematic review data management.

### Quality Appraisal of Studies

The methodological quality of included studies was assessed using the Critical Appraisal Skills Programme (CASP) qualitative checklist [27]. One reviewer (ML) conducted the primary appraisal of each study, while a second reviewer (MEL) independently checked and verified the assessments to ensure accuracy. During this stage, one article was excluded because its methodology and findings were insufficiently reported to allow for reliable appraisal.

### Data Synthesis

A thematic synthesis was conducted following the approach described by Thomas and Harden [23], comprising three overlapping stages: line-by-line coding, development of descriptive themes, and generation of analytical themes.

Verbatim findings from the results sections and participant quotations were imported into NVivo for systematic coding and data management. Two reviewers (MEL and ML) independently read all studies to ensure familiarity with the data, after which meaning units were identified and inductively coded according to their content and relevance to the review aim. Codes were continuously refined and expanded as new studies were added, generating an extensive set of free codes that captured caregivers’ emotions, thoughts, and experiences across the LVAD trajectory.

In the second stage, codes were compared and grouped into preliminary clusters through iterative discussions among all authors (MEL, ML, NPK, TJ, AS, TBG, and BBA). Related codes were then merged into categories and descriptive themes, which were reviewed against the original data to ensure internal consistency and conceptual clarity.

During the final stage, descriptive themes were interpreted at a higher level of abstraction to generate analytical themes. This period, which represents the interpretative core of thematic synthesis, involved moving beyond the manifest content of the original studies to identify new insights and explanatory concepts. The authors independently reflected on the descriptive themes and inferred broader meanings, connections, and implications for understanding caregivers’ experiences. Through iterative group discussions, these interpretations were compared, refined, and integrated to ensure coherence across the dataset. This cyclical process of reflection and reinterpretation continued until the analytical themes were sufficiently abstract to capture and explain all descriptive themes while addressing the overall review aim.

### Ethical Considerations

No ethical approval was required, as the review exclusively synthesised data from previously published research. The study was carried out with a strong emphasis on methodological transparency and scientific integrity, supported by prior protocol registration in PROSPERO (CRD42025619587). To enhance credibility and reduce the risk of bias, multiple stages of the review were completed independently by more than one reviewer. The appraisal of included studies also involved scrutiny of ethical considerations and any reported conflicts of interest.

## Results

### Search Results

**Fig. 1:**
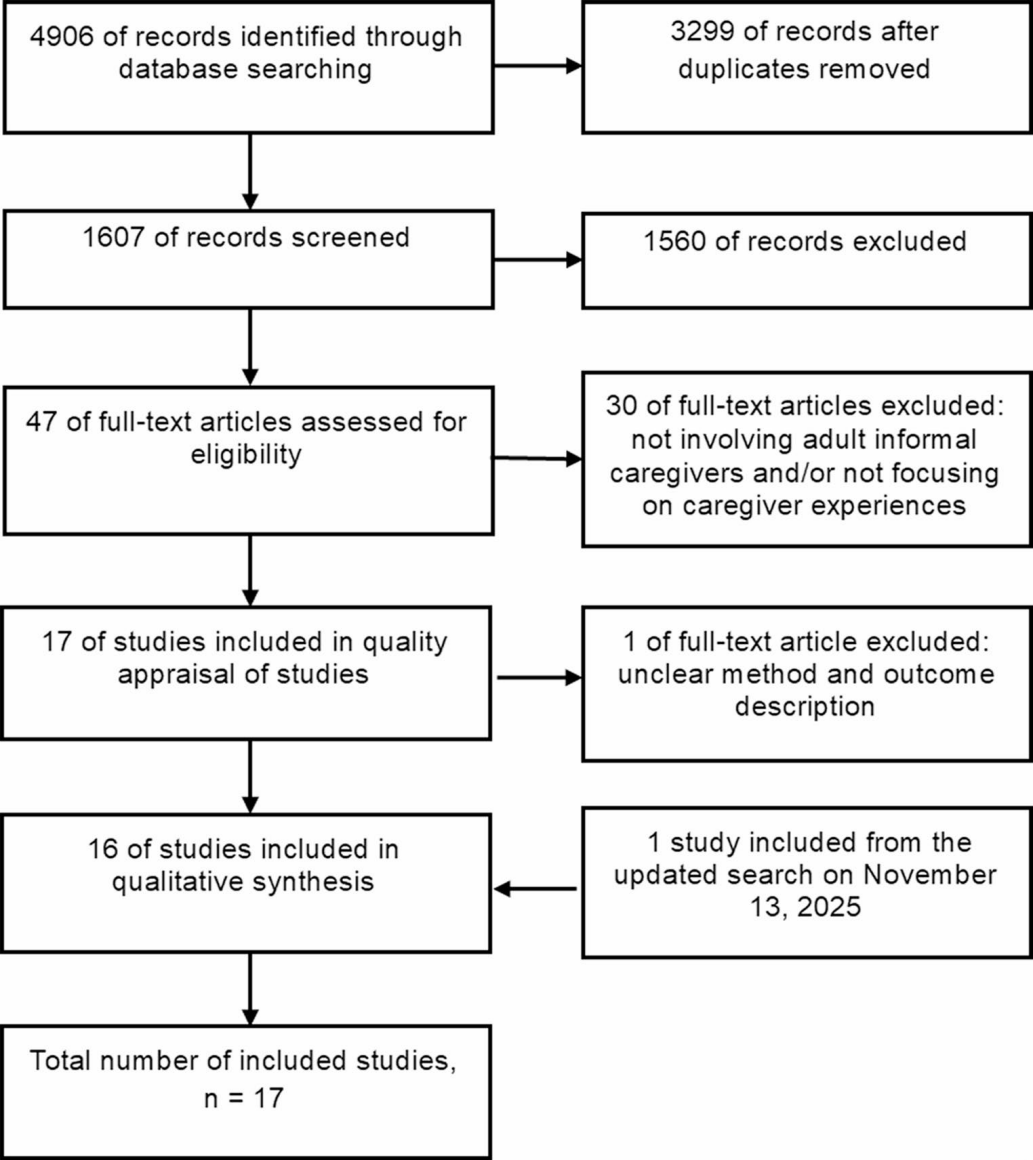
PRISMA flow diagram of study selection process

The database search resulted in 4906 articles. After removing 3299 duplicates, 1607 articles were screened based on title. Of these, 130 abstracts were selected and screened. A total of 47 full-text articles were assessed for eligibility, and 17 studies were included in the quality appraisal. Of these, 16 were finally included in the qualitative synthesis (see Fig. 1). After the updated search, one additional article was included, resulting in a total of 17 included articles (see Supplement File 2).

### Characteristics of Included Articles

The characteristics of the included articles are presented in Table 1. The studies were published between 2015 and 2025 and originate from five countries: the United States (*n* = 12), Singapore (*n* = 2), Canada (*n* = 1), Israel (*n* = 1), and Italy (*n* = 1). Most studies employed qualitative design, including studies using thematic analysis (*n* = 7) and content analysis (*n* = 3), studies using grounded theory (*n* = 2), and one study with a phenomenological-hermeneutic design (*n* = 1). In addition, four studies (*n* = 4) used a mixed-methods approach that included qualitative components. In total, 238 caregivers participated in the included studies.

**Table 1:** Characteristics of the 17 included studies

Author, year, title, country	Aim of the study	Method	Sample size, mean age	Main topic	Results
Abshire et al. 2021, *A picture is worth a thousand words: Exploring the roles of caregivers and the home environment of ventricular assist device patients;* USA	To describe the roles of caregivers and the home environment in VAD patient care and how these influence self-care practices, from the perspective of VAD caregivers.	Qualitative descriptive study using photo-elicitation interviews. Participants (caregivers) were asked to take photos that represent their caregiving experiences. Interviews were conducted to explore the meaning of these images. Data were analyzed using conventional content analysis.	10 caregivers, female 80%, mean age 54.6 years.	Explored the role of informal caregivers and the home environment in the care of patients with ventricular assist devices, using photo-elicitation interviews.	Caregiving evolved as patients gained some independence, but caregivers remained essential. They managed tasks such as driveline care, medication, and personal hygiene. Homes were adapted to support care, mainly in bathrooms and bedrooms. Safety concerns included limited space, infection risk, and improvised adaptations.
Bechthold et al. 2024, *Facilitators and Barriers to Values Discussions Following LVAD Implantation: Perspectives from Diverse Patients and Family Caregivers;* USA	To describe patient- and family caregiver-identified facilitators and barriers to engaging in values discussions with clinicians after LVAD implantation.	Qualitative descriptive approach using one-on-one semi-structured interviews.	27 patients and 21 caregivers, 76% female caregivers, ages 27–76 years.	The study explored the facilitators and barriers to engaging in values discussions with clinicians following LVAD implantation from the perspectives of patients and family caregivers.	Facilitators for communication included close relationships, shared values, clinician-initiated discussions, and dyadic interactions. Barriers included assumptions about shared values, sensitivity around discussions, uncertainty about timing, and poor communication. Both patient- and caregiver-related factors influenced communication.
Bechthold et al. 2024, *“Things That You Thought Mattered*,* None of That Matters”: A Qualitative Exploration of Family Caregiver Values following Left Ventricular Assist Device Implantation;* USA	To understand how caregivers discuss, reflect upon, and act on their values after LVAD implantation.	Qualitative descriptive study using one-on-one semi-structured interviews. Data were analyzed using thematic analysis.	21 caregivers, 76% female, aged 27 to 76 years.	The study explored the values elicitation experiences of family caregivers of individuals with an LVAD in the post-implantation period.	Caregivers re-evaluated their values after LVAD implantation, which guided decision-making and coping. Three themes emerged: using values for strength, changes in relationships and priorities, and expressing goals when relevant to patient care.
Blumenthal-Barby et al. 2015, *Assessment of patients’ and caregivers’ informational and decisional needs for left ventricular assist device placement: Implications for informed consent and shared decision-making;* USA	To explore the decision-making process and informational and decisional needs of patients and their caregivers regarding LVAD placement.	Mixed methods using in-depth, structured interviews and survey instruments with LVAD patients, candidates, and caregivers.	45 participants, including 15 patients, 15 candidates, and 15 caregivers, 66% female. The participants’ ages ranged from 33 to 74 years.	The study investigated the informational and decisional needs of patients and their caregivers regarding LVAD.	Decision-making was experienced as rapid and clinician-driven, with most participants perceiving no real choice. Key information needs focused on lifestyle and technical aspects. Participants identified core values such as life extension, family, and mobility. There was a need for peer support and caregiver involvement, and some showed misunderstanding about transplant eligibility.
Coleman et al. 2025, *Understanding Care Partner Experiences in the First Month After Durable Left Ventricular Assist Device Implantation;* USA	To explore the struggles and support needs of care partners during hospitalization, discharge, and the first month at home after durable LVAD implantation.	Qualitative study using inductive content analysis; semi-structured interviews.	13 care partners; mean age, 85% female, 53 years (range 34–68).	Care partner (caregiver) experiences following durable LVAD implantation.	Caregivers often felt unprepared, overwhelmed, and hypervigilant. They experienced lack of self-care, emotional distress, and isolation. Limited support from healthcare teams and lack of education contributed to these challenges, along with a need for peer support.
DeGroot et al. 2021, *“Talking Around It”: A Qualitative Study Exploring Dyadic Congruence in Managing the Uncertainty of Living With a Ventricular Assist Device;* USA	To explore congruence of VAD patient and caregiver perspectives regarding end of life, definitions of quality of life, and meaning in life while managing the uncertainty of living with a VAD.	Thematic analysis to analyze semistructured qualitative interviews 3 to 12 months after implantation.	10 patient-caregiver dyads caregivers mean age 52.6 years, 80% female.	The communication and congruence between patients with a ventricular assist device and their family caregivers in managing the uncertainty of living with the device.	Patients and caregivers had differing perspectives on uncertainty and end of life. Three themes emerged: uncertainty and worry, end-of-life views, and valuing everyday life and independence. Dyads were more aligned when discussing meaning and quality of life.
Golan et al. 2023, *Couples’ Coping Strategies with Left Ventricular Assist Device Implantation: A Qualitative Dyadic Study;* *Israel*	To formulate a typology of dyadic coping strategies applied by these couples, as unfolded in their mutual and individual subjective experiences.	Qualitative approach using content analysis. Data was collected through in-depth dyadic interviews with couples using a semi-structured interview guide.	17 couples, caregivers ages ranged from 42–75.	The study explored the coping strategies of couples dealing with the implantation of an LVAD.	Couples used different coping strategies to manage fear, illness, and daily life. Five patterns were identified: burden vs. balanced dependence, shared vs. conflicting illness views, acknowledged vs. minimized fear, dyadic vs. individual humor, and adapted vs. avoided intimacy.
Kelemen et al. 2024 *The experiences among bereaved family members after a left ventricular assist device (LVAD) deactivation;* USA	To understand the experiences of family members before, during, and after LVAD deactivation, including their perceptions of engagement with the healthcare team.	Qualitative descriptive study using semi-structured interviews. Data was collected through individual interviews with family members.	11 family members (partners/spouses, parents, children, and siblings) participated.	The study investigated the experiences of bereaved family members of patients who died following LVAD deactivation.	Caregivers’ experiences around LVAD deactivation included hope for survival, importance of communication, spirituality, and absence of physical suffering. Positive relationships with staff and post-death care needs were also highlighted.
Kirkpatrick et al. 2015, *Caregivers and Left Ventricular Assist Devices as a Destination*,* Not a Journey;* USA	To characterize the QOL of caregivers of patients with LVAD-DT and identify burdens and stressors associated with caregiving for these patients.	Concurrent mixed methods design using the City of Hope Quality of Life Family Caregiver instrument, modified for LVAD-DT. Qualitative data were analyzed using a modified grounded theory approach.	42 caregivers (mean age 60, 30-ranged from 78 years), 82% female and 39 patients.	The study explored the quality of life (QOL) of caregivers of patients with Left Ventricular Assist Devices as Destination Therapy.	Caregivers described stress during decision-making, lack of psychological preparation, reduced independence, and constant worry. Psychological and social support were identified as important needs.
Lewis et al. 2021, *The relational dynamics of caregivers of patients with a left ventricular assist device for destination-therapy: A qualitative investigation;* Canada	To investigate the relational dynamics and unique needs of caregivers of patients with DT-LVADs.	Qualitative descriptive secondary analysis using semi-structured interviews with patients, caregivers, and healthcare providers. Thematic analysis.	18 participants, including 3 patients with DT-LVADs, 2 female spouses as caregivers, and 13 healthcare providers.	The study explored the needs and impacts of caregiving for patients with destination-therapy left ventricular assist devices from the shared perspectives of patients, caregivers, and healthcare providers.	Caregiving was shaped by relational dynamics, involving ongoing changes in relationships and daily life. Caregivers navigated needs and impacts within four areas: themselves, the patient, healthcare providers, and the healthcare system.
Magasi et al. 2019, *Preparedness and Mutuality Affect Quality of Life for Patients With Mechanical Circulatory Support and Their Caregivers;* USA	To understand the impact of MCS caregiving on the QoL of patients and caregivers, focusing on preparedness and mutuality.	Secondary analysis of cross-sectional semi-structured qualitative interviews with MCS patients and their caregivers. Thematic analysis was used to analyze the data.	30 patients and 30 caregivers (average age 56 years), 67% female.	The study examined how living with mechanical circulatory support (MCS) affects the quality of life (QoL) of patients and their caregivers through the lens of preparedness and mutuality.	Caregiving had long-term impact on quality of life, with caregivers often entering the role unprepared due to limited decision time. Themes included forced choice, adjustment, relationship changes, strain, and burden. Preparedness, mutuality, and support networks were central to the caregiving experience.
McIlvennan et al. 2015, *Decision-making for destination therapy left ventricular assist devices: implications for caregivers;* USA	To understand the caregivers’ experiences and identify their needs related to decision-making surrounding DT LVAD.	Qualitative descriptive study using in-depth, semi-structured interviews with caregivers of patients currently living with a DT LVAD, caregivers of patients who had died with a DT LVAD, and caregivers of patients who had refused a DT LVAD.	17 caregivers (including 10 caregivers of patients living with DT LVAD, 6 caregivers of patients who had died with DT LVAD, and 1 caregiver of a patient who had declined DT LVAD). The age ranged from 35 to 79 years. 94% female.	The study explored the decision-making process and experiences of caregivers of patients considering destination therapy left ventricular assist devices (DT LVADs).	Caregivers experienced decision-making as complex and emotionally challenging. Three domains emerged: decision context (hope vs. reality), decision process (balancing patient wishes and survival), and decision outcome (gratitude vs. burden)
McIlvennan et al. 2016, *Bereaved Caregiver Perspectives on the End-of-Life Experience of Patients With a Left Ventricular Assist Device;* USA	To understand the experiences of bereaved caregivers and patients at the end of life who have an LVAD.	Qualitative descriptive study using in-depth, semi-structured interviews with bereaved caregivers of patients with an LVAD.	8 bereaved caregivers (median age 64, range 54–71), 80% female.	The study explored the experiences of bereaved caregivers of patients with an LVAD at the end of life.	Caregivers described confusion in the final stage of care. Themes included uncertainty about the dying process, legal and ethical aspects, and fragmented palliative and hospice care.
McIlvennan et al. 2021, *Stress and Coping Among Family Caregivers of Patients With a Destination Therapy Left Ventricular Assist Device: A Multicenter Mixed Methods Study;* USA	To characterize longitudinal stress, identify predictors and correlates of stress, and explore coping processes among caregivers of patients with DT LVADs.	Sequential exploratory mixed-methods study, with qualitative component: Semi-structured interviews guided by the Transactional Model of Stress and Coping. Thematic analysis of caregiver experiences.	25 caregivers in qualitative analysis (mean age 66,1), 90% female.	Stress and coping mechanisms among family caregivers of patients with a destination therapy left ventricular assist device.	Caregivers experienced stress related to lack of preparedness, unique caregiving challenges, and physical and emotional demands. Emotion-focused coping was commonly used.
Neo et al. 2020, *Life Beyond Heart Failure-What Are the Long-Term Challenges*,* Supportive Care Needs*,* and Views Toward Supportive Care of Multiethnic Asian Patients With Left Ventricular Assist Device and Their Caregivers?;* Singapore	To explore the long-term challenges, supportive care needs, and perceptions toward supportive care among multiethnic Asian LVAD patients and their caregivers.	Qualitative study using grounded theory. Semi-structured interviews with thematic analysis.	30 patients and 11 caregivers (mean age 55,5), 64% female caregivers.	Long-term challenges and supportive care needs of multiethnic Asian patients with LVADs and their caregivers.	LVAD implantation led to long-term changes across physical, financial, social, and emotional domains, with ongoing losses over time. Caregivers and patients emphasized mutual support, connectedness, and the need for holistic care.
Neo et al. 2021, *Lived Experiences and Long-Term Challenges and Needs of Asian Left Ventricular Assist Device Caregivers;* Singapore	To explore the long-term challenges and needs of caregivers of patients living with LVADs in an Asian healthcare setting, with the goal of informing culturally relevant supportive care interventions.	Qualitative study using grounded theory. Semi-structured interviews with thematic analysis.	11 caregivers, 64% female caregivers. Mean age 55.5 years).	Long-term caregiving experiences, challenges, and needs of Asian caregivers of patients with LVADs.	Caregivers experienced long-term, multifaceted challenges across physical, financial, emotional, and social domains. Coping was influenced by expectations, cultural factors, and limited social support. Key needs included perspective shifts, improved communication, advocacy, and holistic long-term care.
Rapelli et al. 2023, *“The heart in a bag”: The lived experience of patient-caregiver dyads with left ventricular assist device during cardiac rehabilitation;* Italy	To explore the lived experiences, emotional responses, and psychological needs of patients with LVAD and their caregivers during cardiac rehabilitation, prior to discharge and home management.	Qualitative study using a phenomenological hermeneutic approach. Semi-structured, face-to-face interviews. Thematic analysis with dyadic comparison.	6 patient-caregiver dyads (12 individuals), caregivers mean age 51.1 years (range: 49–71).	Lived experiences and psychological adjustment of patient-caregiver dyads with LVAD during cardiac rehabilitation.	Caregivers described living between life and death, managing fear and uncertainty, and adapting to the device. Caregiving involved both shared support and burden, while also feeling passive and dependent on external factors.

### Synthesis Results

In the thematic synthesis, eleven descriptive themes were identified, generating four analytical themes describing caregivers’ experiences of living with an adult supported by an LVAD. Using an event-timeline approach, these analytical themes, *Before LVAD implantation*, *Early post-LVAD*, *Later post-LVAD*, and *End of the LVAD journey*, reflect the evolving and dynamic nature of caregiving across the LVAD trajectory.

**Fig. 2:**
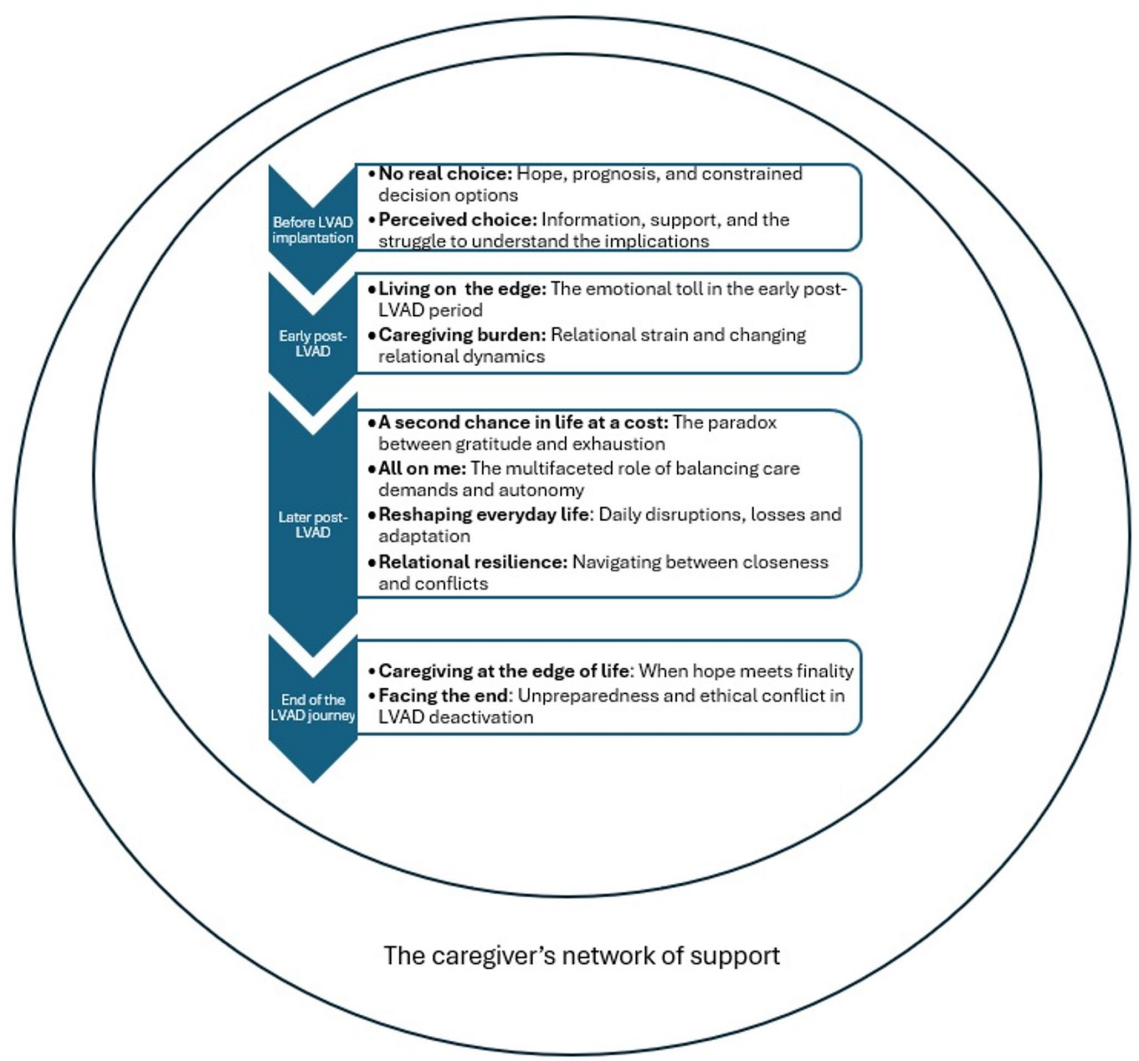
An illustration of the caregivers’ situation during the LVAD trajectory

In addition to these time-dependent analytical themes, an overarching analytical theme, *The caregiver’s network of support*, was identified. This theme captures caregivers’ experiences of both receiving and providing support through formal and informal networks throughout the LVAD journey. It represents a continuous and cross-cutting aspect of caregiving that extends across the entire trajectory rather than being confined to a specific time point (see Fig. 2).

#### Before LVAD implantation

This first analytical theme begins before LVAD implantation and consists of two descriptive themes, *No real choice* and *Perceived choice.* Together, these descriptive themes show how caregivers experienced this period as emotionally demanding and shaped by limited choice. They faced hope mixed with the reality of a poor prognosis, emotional strain, and a sense that no meaningful alternatives were available, while also trying to navigate the decision under time pressure, with limited autonomy, and varying levels of informational and emotional support from healthcare professionals.

##### No real choice: Hope, Prognosis, and Constrained Decision Options 

While some caregivers perceived that they had a choice, others felt that there was no real alternative, as the only other option was death [28–30]. The LVAD was seen by caregivers as a possibility of extending the life of the person they care for, in which they placed their hope [31]. However, some caregivers experienced an underlying tension between this hope for the future and the reality with a very poor prognosis [29]. The LVAD as a BTT raised issues related to organ donation, and caregivers knew the wait for a new heart could be long, or in vain [32].

Caregivers described the pre-implantation period as one filled with uncertainty and considerable emotional strain. Some had to make rapid decisions when the patients were critically ill, often without fully processing all the information provided. Many wished for more time to align the decision with the person’s they care for wishes. The pre-operative period was characterized by anxiety, vulnerability, and unmet expectations, with some caregivers later expressing regret [28, 29, 33].

This gave rise to feelings of ambivalence, while they understood the necessity of the LVAD, they also reported struggling to come to terms with it, describing the need to get used to and ultimately accept the device as part of their new reality [32].

##### Perceived Choice: Information, Support, and the Struggle to Understand the Implications

The decision-making process was described as a brief yet challenging period. Some felt “backed against the wall” [30], which led them to accept the LVAD without fully contemplating the risks of surgery, potential complications, or the long-term burdens of DT-LVAD [29].

The medical team played a key role in their decision-making process. The support of healthcare professionals was a source of relief for caregivers, who believed that the guidance offered was likely aligned with their best interests. However, there was also frustration about the urgency of the decision and the perceived pressure to proceed with a DT LVAD [29].

For some caregivers, the information and preparation provided were perceived as sufficient to meet the practical responsibilities of LVAD care [28, 33]. However, many reported insufficient preparation for the psychological and social challenges, and some felt unprepared overall due to limited hands-on training and reliance on verbal information [30, 34].

#### Early Post-LVAD

This analytical theme consisted of two descriptive themes, *Living on the edge* and *Caregiver burden*. Together, the descriptive themes show how caregivers experienced the early post-LVAD period as emotionally and relationally demanding. They faced fear, uncertainty, and hypervigilance while carrying the heavy responsibility of supporting a patient dependent on a life-sustaining device, alongside relational strain, personal exhaustion, and the challenges of navigating shifting roles and increasing dependency.

##### Living on the Edge: Emotional Toll in the Early Post-LVAD Period

The immense responsibility associated with caregiving after LVAD implantation left many feeling overwhelmed, describing the experience as both burdensome and emotionally exhausting [32]. Feelings of fear and uncertainty were commonly reported immediately after the implantation. Many described the anxiety of having to rely on a machine to keep the person they care for, alive [35–37], and how the device led to such drastic changes in the person’s they care for behaviour and personality that they sometimes no longer recognized them [38]. At the same time, caregivers were expected to manage their own emotions and help calm the patient [32, 39].

A constant state of hypervigilance, caused by potential LVAD complications, was described by caregivers, with the transition from hospital to home considered the most stressful period [30, 33–35, 37, 39]. They reported role changes, loss of control, and emotional strain [38]. This lack of preparedness fostered sustained hypervigilance that, over time, accumulated into overwhelming strain and a sense of invisibility, as caregivers’ own needs and identities receded behind the imperative to keep the patient alive [35]. Caregivers used various coping strategies to manage emotional stress and the sense of lost control, including engaging in hobbies, spending time outdoors, using humour to reframe challenging situations, and taking one day at a time to avoid becoming overwhelmed by an uncertain future. These strategies helped them maintain moments of normalcy and reduce the pressure of constantly thinking ahead [36, 40].

##### Caregiving Burden: Relational Strain and Changing Relational Dynamics

The patient was often focused on his own transition, emotions, and needs leading to a sense of detachment, where caregivers felt the patient did not fully appreciate the realities of their daily life and challenges [38]. The caregiving experience was frequently associated with relationship strain, perceived burden, and a lack of mutual understanding and intimacy between patient and caregiver [30]. This often intensified the caregiver’s sense of burden, particularly when patients did not share the same preferences or wishes as the caregiver, leading to tension and relationship difficulties [29], while also contributing to the patient’s perceived loss of autonomy, as patients felt like a burden, became increasingly dependent, and observed their caregivers becoming drained, unprepared, and exhausted [32].

Moreover, the lack of preparedness for the magnitude and complexity of their role, contributed to a constant state of alertness, where caregivers closely monitored the patient and device, often at the expense of sleep, employment, and personal well-being [35]. Many caregivers reported neglecting their own health while caring for their relative, which led to physical and emotional consequences such as stress, weight loss, sleep disturbances, difficulty concentrating, and an inability to attend their own medical appointments [34].

#### Later Post-LVAD

This analytical theme consists of four descriptive themes, *A second chance to live at a cost*, *All on me*, *Reshaping everyday life* and *Relational resilience*, describing how caregivers navigated gratitude and exhaustion, managed extensive care responsibilities while balancing autonomy, adjusted everyday life to substantial disruptions and managed relationship dynamics involving both closeness and tension.

##### A Second Chance to Live at a Cost: The Paradox Between Gratitude and Exhaustion

The LVAD was described as both a source of hope and a “second chance at life,” motivating caregivers to cherish meaningful moments with the person they care for despite ongoing challenges [30, 32, 40]. The implantation often prompted reflection on relationships and life priorities, with many caregivers placing the patient and device above all else, sometimes at the expense of their own health and well-being [34, 37, 41]. While this deepened sense of purpose strengthened commitment, it also contributed to stress, sleep problems, and caregiver burnout [34, 37].

However, caregivers also experienced increasing physical strain during this period as their responsibilities affected their independence and daily routines. For some, the demands of caregiving led to physical exhaustion and reduced capacity for self-care, while the presence of the LVAD, although life-prolonging for the patient, contributed to ongoing physical burden for the caregiver [30, 32]. Difficulties related to the patient’s adaptation to the LVAD intensified the caregiver’s physical workload and sense of practical responsibility [32, 34].

Faith and spirituality emerged as significant coping mechanisms, offering comfort, meaning, and resilience in times of uncertainty [39, 40]. Caregivers frequently interpreted their experiences through a spiritual lens viewing their role as a test or lesson, which often deepened their sense of gratitude and acceptance [32, 37].

##### All on Me: The Multifaceted Role of Balancing Care Demands and Autonomy

Daily life after LVAD implantation was, for many caregivers, characterized by extensive responsibilities such as medication management, medical coordination, and regular VAD dressing changes [42]. The accumulation of medical and nonmedical tasks profoundly affected their independence, social life, and relationships, particularly during the early post-hospitalization period, which was often described as overwhelming [32, 42]. While some gradually developed routines, others remained the sole person responsible for care, leading to exhaustion, frustration, and feelings of entrapment [28, 32, 36]. Physical and emotional strain was amplified by the patient’s dependency and by high-stress tasks such as sterile dressing changes [34, 39].

In addition to practical responsibilities, caregivers provided psychological support and, at times, took an active role in decision-making [32]. Some accepted this as part of their relationship, while others expressed frustration over insufficient preparation and lack of recognition for their role [33]. Balancing support with respect for patient autonomy was central, with caregivers initially supervising closely before gradually stepping back to foster independence [40, 42].

##### Reshaping Everyday Life: Daily Disruptions, Losses and Adaptation

Providing care for a person with an LVAD involved substantial losses, lifestyle disruptions, and a reorientation of daily priorities, with the patient’s well-being becoming central to nearly all decisions [36, 40, 42]. Everyday routines, such as travel, meal preparation, and home organization, were continually adjusted to meet the patient’s needs and the technical demands of device management. Bedrooms frequently became multifunctional spaces for medical equipment and supplies, and furniture and sleeping arrangements were modified to ensure safety, accessibility, and proximity to power sources [36, 42]. Although often small, these adaptations had major effects on daily functioning and household dynamics.

LVAD-related tasks soon became routine but demanded constant vigilance, including battery changes, alarm management, and protecting the device during showers [30, 36, 39]. Fears of emergencies in public sometimes led to reduced social engagement, withdrawal, and isolation, particularly among sole caregivers or those balancing additional family and financial responsibilities [39, 40]. A limited understanding of LVAD care among extended family and friends is often further restricted practical support [37].

Caregivers frequently sacrificed social and leisure activities, avoided leaving the patient alone, and restricted travel, contributing to feelings of confinement and loss of autonomy that often intensified over time [30, 34, 37, 43]. The later post-LVAD period was described as an emotional “rollercoaster,” in which recurring periods of stress and fatigue accumulated alongside gradual adaptation, while a persistent state of vigilance and readiness remained [30, 32]. Financial strain and employment instability added further challenges, as some caregivers increased work hours to compensate for lost patient income, while others faced lack of understanding from employers or social institutions unfamiliar with LVAD therapy [30, 37, 39].

Sexual and intimate relationships also required adjustment. Many couples shifted toward non-sexual forms of closeness, while sexual activity was reduced or avoided due to concerns about safety, age, or pre-existing changes in sexual habits [36, 39]. For some, the loss of sexual intimacy and fertility contributed to emotional distress and altered future life planning, even when these changes were ultimately accepted as part of the LVAD journey [33, 37].

##### Relational Resilience: Navigating Between Closeness and Conflicts

Closeness in the relationship with the person they care for was often described by caregivers as a key factor in coping with the long-term demands of life with an LVAD [39, 43]. Many attributed their strong bond to a long history of shared experiences and reciprocal support, which allowed them to adapt more effectively to caregiving challenges. The interdependence fostered through the caregiving role, along with the intimate nature of daily care, was often perceived as strengthening the relationship. Moreover, the LVAD was seen, not only as a life-sustaining intervention, but also as an opportunity to spend more meaningful time together, reinforcing mutual reliance and emotional closeness [30, 39, 43].

Another facilitator for coping was the increase in meaningful dialogue and values-based conversations with the patient, often focusing on gratitude for extended time together, physical limitations, and shared adjustments in daily life [43]. While some caregivers reported open communication prior to LVAD implantation, others noted that the device created new opportunities for deeper discussions, fostering mutual understanding and emotional closeness [36].

However, LVAD care also led to challenges and conflict, particularly regarding practical tasks and healthcare decisions. Disagreements often left caregivers feeling uncertain and emotionally overwhelmed, with tensions arising from individual personalities, differing accounts of illness, and contrasting perspectives on the patient’s condition and treatment [36, 37, 40, 43]. Practical aspects of device management sometimes generated tension, as caregivers balanced their own emotional responses with the patient’s needs while navigating cultural or privacy considerations that limited disclosure to others [36, 37].

#### End of the LVAD Journey

This analytical theme consists of two descriptive themes, *Caregiving at the edge of life*, and *Facing the end*,* which together* describe how caregivers confronted existential distress at the end of life, navigating fear of death, profound uncertainty about the future, unpreparedness for the dying process, and moral and emotional conflict surrounding LVAD deactivation.

##### Caregiving at the Edge of Life: When Hope Meets Finality

Thoughts about death represented a complex and emotionally charged aspect of existential distress for caregivers. For some, the topic was overwhelming, leading to avoidance of conversations about death, often linked to the deep emotional bond with the patient and fear of life without them [41]. Emotional responses were influenced by past experiences, visual reminders, and personal coping styles, with some caregivers unsettled by hospital encounters and others drawing on spirituality or faith to frame end-of-LVAD events as part of a higher plan, easing the burden of decision-making [31, 41]. Despite avoidance, caregivers valued assurance that the person they care for would be comfortable and free from pain after LVAD deactivation, which provided emotional relief and aided coping [31].

Another significant source of distress was uncertainty about the future. For caregivers of patients on BTT, uncertainty centred on the timing and outcome of transplantation, compounded by awareness of the LVAD’s limited lifespan and pressure to “make the most” of remaining time [37, 39]. For caregivers of DT patients, distress was more often connected to confronting death and navigating the emotional challenges of device deactivation [37, 44]. Some caregivers coped by focusing on day-to-day life, though this reflected unresolved underlying uncertainty [41]. Confusion about the dying process, particularly when patients chose to discontinue support, was common, as caregivers often did not know how or when death would occur after deactivation [44].

##### Facing the End: Unpreparedness and Ethical Conflict in LVAD Deactivation

Many caregivers reported being unprepared for the patient’s trajectory toward end of life, and felt overwhelmed by the complexities and emotional intensity of the dying process in the context of LVAD support [31, 44]. This unpreparedness was partly rooted in patients’ histories of severe illness followed by recovery, leading caregivers to hold onto hope even during final hospitalizations, and experience shock when deterioration proved irreversible [31]. In the final days and weeks, caregivers reported heightened anxiety, confusion, and uncertainty about what to expect clinically and practically, particularly if death occurred outside the hospital or emergency personnel lacked LVAD-specific knowledge [31, 44].

Even the deactivation process itself caused significant distress. The legal and ethical dimensions of turning off the device often left caregivers feeling unprepared, confused, and morally conflicted, with a few even interpreting it as suicide or murder. Religious beliefs sometimes reinforced the view that such decisions should be left to a higher power rather than humans [31, 44].

#### The Caregiver’s Network of Support

This overarching analytical theme described how support networks were essential throughout the LVAD trajectory, helping caregivers manage emotional and practical demands and reducing overreliance on single individuals [30, 40]. Emotional support, particularly early psychological assistance, was highly valued and highlighted the need for holistic care that addressed caregivers’ fears and future uncertainty [32, 35, 37]. Family commitment, grounded in values such as responsibility, faith, and loyalty, strongly motivated caregiving and often involved redistributing roles or training others to ensure continuity of care [39, 40]. Peer support from other LVAD caregivers provided reassurance and practical guidance [35], though concerns about privacy and exposure to others’ grief sometimes limited participation [33, 39].

Limited community awareness frequently led to stigma, misunderstandings, and challenges in work and social reintegration, requiring caregivers to repeatedly explain the LVAD, which contributed to exhaustion and social withdrawal [37, 39]. Many caregivers developed strong bonds with the LVAD team, especially coordinators, whose personalized, consistent support fostered trust and made caregivers feel understood [43]. However, frequent staff turnover disrupted these relationships and made it more difficult to maintain continuity and openness over time [28, 43].

Caregivers often felt overlooked in clinical encounters, noting that care discussions focused almost exclusively on the patient despite caregiving’s substantial impact on their own physical and emotional well-being [38, 43]. This lack of inclusion reinforced feelings of invisibility, even though caregivers played a critical role in ongoing LVAD management.

During the end-of-LVAD period, supportive communication from LVAD and palliative care teams was highly valued, helping caregivers understand the dying process, align decisions with patient values, and feel emotionally supported [31, 44]. Continuity of care, presence during the final hours, and post-loss contact were described as meaningful sources of comfort, supplemented by wider networks such as friends, counsellors, and faith communities [31].

In contrast, some caregivers reported unclear or inconsistent information, leading to confusion and emotional strain [31, 44]. Communication discrepancies between clinical teams created conflicting impressions of the patient’s condition. Feelings of abandonment were also described during transitions to hospice or community care, where professionals were often unfamiliar with LVAD-specific needs—forcing caregivers to guide staff themselves. These gaps underscore the need for consistent, knowledgeable, and compassionate support across all settings [44].

## Discussion

The study highlights the multifaced trajectory experienced by caregivers to patients with an LVAD, showing that they face substantial and multidimensional burdens across the entire caregiving journey. When the LVAD extends patient survival and improves quality of life, it simultaneously places significant emotional, practical, and existential demands on caregivers. These challenges are dynamic rather than static, shifting from acute emotional strain during the decision-making and early recovery, to long-term role overload, relationship strain, and to profound existential distress in the end-of-LVAD period.

This trajectory demonstrates that caregiver support cannot rely on a one-size-fits-all model. Caregivers cannot be understood as a homogeneous group; rather, they include partners, siblings, parents, and friends, each entering the LVAD journey with different relational histories, emotional bonds, and expectations. These differing relationships shape both how they experience the patient’s illness and the type of support they themselves require. As highlighted by Alleman et al. [45], caregivers’ roles, motivations, and coping capacities vary substantially depending on their connection to the patient, reinforcing the need for tailored and relationship-sensitive support. Instead, interventions must be tailored to the dynamic challenges and needs that emerge at different stages of the LVAD trajectory. To mitigate these challenges, structured caregiver training programs, psychological support, and access to social and practical interventions are essential. However, the results of our systematic review show that such structured and time-specific support is currently insufficient or inconsistently provided.

In the period before LVAD implantation, caregivers experience intense emotional strain and uncertainty, alongside a recurring sense of having “no real choice” when confronted with the need to make decisions about LVAD therapy. Studies in our review show that hope for survival, limited time, and poor prognosis often drive decisions, leading to decisional conflict, anxiety, and insufficient understanding of risks and long-term caregiving demands. These findings mirror the DECIDE-LVAD trial, where caregivers entered the process with high stress, limited knowledge, and decisions not always aligned with their values [46]. Although DECIDE-LVAD improved value–choice alignment, it offered limited preparation for the emotional and practical challenges awaiting caregivers [47]. ENABLE-LVAD provides a complementary pre-implant psychoeducational approach that strengthens communication, coping, and emotional readiness [48]. Together, evidence indicates that early caregiver involvement can enhance preparedness and confidence [49], yet existing support remains insufficient. Our synthesis highlights the need for comprehensive, tailored pre-implant strategies, including decision aids, structured shared decision-making, and targeted preparatory interventions, to ensure caregivers are adequately informed, emotionally supported, and better prepared for the demands of LVAD care [50].

In the early post-LVAD period, caregivers face intense emotional strain marked by fear of complications, hypervigilance, and a profound sense of responsibility for the patient’s survival. Much of this burden stems from constant monitoring of alarms, batteries, and driveline integrity, alongside worries about device malfunction during everyday activities such as bathing, sleeping, and travelling, which reduces their sense of safety. These device-related concerns heighten anxiety and place caregivers in the dual role of managing both the patient’s distress and their own fear [51]. Our review further shows that drastic behavioural changes in the patient, reduced emotional reciprocity, and persistent vigilance contribute to caregiver burden, relational tension, and neglect of their own well-being. Distress, sleep disruption, social limitations, and impaired self-care often emerge early in the caregiving trajectory and remain moderate to severe long after discharge, with many caregivers reaching levels that warrant psychological support [7]. Since LVAD care is inherently dyadic, caregiver well-being directly affects patients’ capacity for self-care, and dyadic strategies such as shared coping and joint management are essential for successful adaptation [52]. Overall, the early post-LVAD period clearly demands comprehensive, continuous, and dyad-oriented interventions to address hypervigilance, emotional exhaustion, relationship strain, and the significant demands of the transition to home.

In the later post-LVAD period, caregivers continue to experience substantial and multifaceted burdens despite more established routines. Gratitude for the patient’s “second chance” coexists with persistent physical strain, sleep problems, loss of autonomy, and chronic role overload. Many caregivers manage complex medical and household responsibilities, which can lead to exhaustion, social isolation, financial strain, and declines in their own health and well-being. These challenges have been shown to persist for months and even years after implantation, underscoring the need for ongoing, individualized support [9]. Caregivers also report difficulty retaining essential LVAD skills over time, highlighting the importance of accessible follow-up and periodic refresher training to maintain confidence [53]. Practical strategies such as structured retraining, simulation-based refreshers, and home-based follow-up may strengthen competence and reduce device-related anxiety, supported by evidence that simulation-based mastery learning improves VAD management skills [54]. Additionally, relational impacts remain prominent: while some dyads develop greater closeness, others struggle with conflict, reduced intimacy, and communication challenges. These findings align with research showing persistent relational strain after advanced cardiac events, suggesting that couples-based counseling and facilitated values-based discussions may enhance relational resilience [55]. Financial and occupational disruptions further indicate the need for social work support to assist caregivers in navigating employment issues, benefits, and community resources.

In the end-of-LVAD period, caregivers experience profound existential distress, anticipatory grief, and moral uncertainty, highlighting the need for proactive, targeted support. Decisions about LVAD deactivation often generate confusion, fear, and ethical conflict, which can be mitigated through early, structured, and value-based conversations about end-of-LVAD options [56]. Timely discussions that clarify the clinical and emotional implications of device withdrawal may reduce guilt, moral distress, and decisional burden. Early integration of palliative care in the LVAD trajectory improves preparedness, symptom management, and family support as the patient nears the end of life [57]. Caregivers’ reports of shock, unpreparedness, and existential turmoil during the dying process underscore the importance of interventions addressing meaning-making, spiritual needs, and anticipatory grief. Approaches grounded in advance care planning, exploration of preferences, and strong palliative care involvement can help caregivers make sense of the final period and cope more adaptively [58]. Overall, end-of-LVAD support for LVAD caregivers must be comprehensive - encompassing ethical guidance, psychological and spiritual care, and structured palliative input, to reduce suffering and assist families through the final stages of the LVAD journey [59].

However, beyond these time-specific needs, our findings also highlight the continuous importance of social, emotional, and professional support, as reflected in the overarching analytical theme, T*he caregiver’s network of support.* Caregivers consistently emphasized that their ability to sustain LVAD care depended not only on structured period-related interventions but also on reliable support networks throughout the entire trajectory. Emotional support from family, peers, and psychological services helped them navigate uncertainty and fear, while practical assistance reduced the risk of overburdening a single individual [18]. This is consistent with recent findings showing that perceived social support is strongly associated with improved psychological outcomes in LVAD populations, including lower anxiety and depression and higher quality of life, underscoring the importance of developing multi-professional social support interventions [60]. Supportive relationships with LVAD teams were also crucial for building caregivers’ confidence and maintaining continuity in care, although disruptions in communication or staff turnover sometimes weakened this sense of security. Many caregivers expressed a desire to be acknowledged as partners in care, noting that their own well-being was often overlooked despite their central role in sustaining day-to-day management. Toward the end of the LVAD journey, consistent and compassionate communication from LVAD and palliative care teams was especially valued, helping caregivers understand the situation and align decisions with the patient’s values.

These findings underscore that caregivers require reliable, coordinated support that extends beyond individual time periods of the LVAD trajectory. They also illustrate the essential role of caregivers in ensuring safe and sustainable LVAD management [61], a dependency that is formally embedded clinical practice, through the requirement of a designated support person to enable home-based LVAD care [62]. This reliance demonstrates that caregiving is not merely an adjunct to treatment, but an embedded structural requirement within LVAD therapy. However, this dependency also raises critical questions about how well current systems prepare, support, and protect the individuals who are expected to shoulder such responsibility. Importantly, these expectations are unlikely to be experienced uniformly among caregivers, suggesting the need to consider how individual circumstances and caregiving roles shape support needs.

The caregiving roles described in this review reflect well-established gendered patterns in informal caregiving. Women constituted the majority of caregivers and are known to assume a disproportionate share of intensive and personal caregiving tasks, spend more hours providing care, and experience greater cumulative burden than men [63, 64]. Further, evidence shows that female caregivers are more vulnerable to long-term psychosocial consequences, including depression and functional difficulties, and receive less practical support than male caregivers [63, 65]. In the context of LVAD therapy, where caregiving is continuous, technologically complex, and formally required, these gendered inequities may be amplified. Treating caregivers as a homogeneous group risks overlooking women’s specific challenges, underscoring the need for tailored, gender-sensitive support to protect caregiver well-being and ensure safe and sustainable LVAD management [61, 62]. These findings highlight that caregiver support must be sustained, coordinated, and responsive to individual and relational differences across the LVAD trajectory, providing a clear rationale for re-examining how current care models and guidelines address the needs of informal caregivers. This sets the stage for a broader discussion on how existing guidelines frame caregiver involvement, and how these expectations align with the structured support available to them.

### Strengths and Limitations

A major strength of this systematic review is the use of thematic synthesis to integrate qualitative evidence on informal caregivers’ experiences of everyday life when supporting adults with advanced heart failure treated with an LVAD. Following the framework by Thomas and Harden [23] enabled a transparent and systematic analytical process, with a clear distinction between descriptive and analytical themes, thereby enhancing conceptual clarity and methodological rigour.

Methodological transparency was further strengthened through adherence to ENTREQ and PRISMA guidelines and prior registration of the review protocol in PROSPERO, reducing the risk of selective reporting. The comprehensive search strategy, developed and peer reviewed by medical librarians in accordance with the PRESS guideline and conducted across six major databases, increased the likelihood of identifying relevant studies. In addition, investigator triangulation, with multiple reviewers involved in screening, data extraction, quality appraisal, and synthesis, enhanced credibility and reduced interpretative bias.

Several limitations should also be acknowledged. Variability in study contexts, healthcare systems, caregiving roles, and caregiver definitions across the included studies may have influenced the comparability and transferability of the findings, including potential cultural and contextual differences. In addition, the synthesis relied on secondary interpretations of primary qualitative data and was dependent on the reporting quality of the original studies. Although reflexive discussions were used to mitigate subjectivity, alternative interpretations remain possible, and insight into contextual and longitudinal aspects of caregiving may therefore be limited.

A further limitation relates to the cultural and contextual distribution of the included studies. The majority were conducted in North America, which should be considered when interpreting the transferability of the findings to other cultural and healthcare settings. While evidence from outside North America (e.g. Asia, Europe, and the Middle East) contributed to similar overarching themes, such as caregiver burden, emotional strain, and role adaptation, some contextual differences were observed. In particular, findings from Asian settings highlighted the influence of cultural and religious norms, including caregiving as a moral or familial obligation, the role of spirituality in coping, and the presence of social stigma. In contrast, evidence from European and Middle Eastern contexts placed greater emphasis on dyadic coping processes, relational dynamics, and shared meaning-making within patient-caregiver relationships. These findings suggest that although core caregiving challenges may be consistent across contexts, the ways in which they are experienced and managed are shaped by cultural and institutional factors.

## Conclusion

This systematic review shows that informal caregivers’ experiences of everyday life when supporting an adult with an LVAD are dynamic and evolve across the entire care trajectory. From emotionally constrained decision-making before implantation to long-term caregiving and end-of-LVAD journey care, caregivers face substantial and cumulative emotional, practical, and relational demands. Across all periods of the LVAD journey, caregivers both receive and provide support through formal and informal networks, highlighting support as a continuous and integral component of caregiving rather than one confined to specific time points. While caregivers demonstrate adaptability, commitment, and resilience over time, the ongoing demands of caregiving profoundly affect daily life, relationships, and well-being. These findings underscore the importance of sustained, structured, and knowledgeable support for informal caregivers across the entire LVAD pathway, aligned with their evolving needs and roles.

### Relevance to Clinical Practice

This review highlights the everyday challenges faced by informal caregivers of adults living with an LVAD and underscores the essential role caregivers play in long-term heart failure management. The findings emphasise the importance of targeted support tailored to caregivers’ specific informational, emotional, and practical needs throughout the LVAD trajectory. Incorporating caregivers’ perspectives into clinical nursing practice can guide the development of structured education, psychosocial interventions, and follow-up strategies, thereby strengthen caregiver preparedness and promoting person- and family-centred LVAD care.

## Key References

- Magid, M., et al., *The Perceptions of Important Elements of Caregiving for a Left Ventricular Assist Device Patient: A Qualitative Meta-Synthesis.* J Cardiovasc Nurs, 2016. 31(3): p. 215 − 25.
  - ○ This meta-synthesis identifies LVAD caregiving as an intense and evolving process marked by significant burden and the need for ongoing adaptation. It highlights gaps in understanding caregivers’ experiences across the full trajectory, reinforcing the need for comprehensive, longitudinal perspectives such as those addressed in this review.

- Magasi, S., et al., *Preparedness and Mutuality Affect Quality of Life for Patients With Mechanical Circulatory Support and Their Caregivers.* Circ Cardiovasc Qual Outcomes, 2019. 12(1): p. e004414.
  - ○ This qualitative study highlights the burden placed on MCS caregivers and shows that device implantation is often experienced as a forced choice with no real alternative. It underscores the psychological impact of entering caregiving under constrained circumstances and the need for better preparation and shared decision-making.

- McIlvennan, C.K., et al., *Stress and Coping Among Family Caregivers of Patients With a Destination Therapy Left Ventricular Assist Device: A Multicenter Mixed Methods Study.* Circ Heart Fail, 2021. 14(10): p. e008243.
  - ○ This mixed-methods study provides important insight into both the measurable and lived experience of caregiver stress in LVAD care. By combining longitudinal quantitative data with qualitative interviews, it highlights how stress is closely linked to preparedness, emotional burden, and patient wellbeing, underscoring the need for better caregiver support before and after implantation.

- Bidwell, J.T., K.S. Lyons, and C.S. Lee, *Caregiver Well-being and Patient Outcomes in Heart Failure: A Meta-analysis.* J Cardiovasc Nurs, 2017. 32(4): p. 372–382.
  - ○ This meta-analysis highlights the clinical importance of the patient–caregiver dyad, showing that caregiver strain is closely linked to patient outcomes. It supports the need for dyadic interventions to improve symptom management and quality of life.

## Supplementary Information

Below is the link to the electronic supplementary material.

ESM 1 (DOCX 26.6 KB)

ESM 2 (DOCX 17.9 KB)

## Data Availability

Data sharing is not applicable to this article as no datasets were generated or analysed during the current study.
