# Supplementary material for: The Dynamic Nature of Caregiving in Advanced Heart Failure With Left Ventricular Assist Device Support: A Systematic Review and Thematic Synthesis
Source: Curr Heart Fail Rep. 2026 Apr 30;23(1):19. doi: 10.1007/s11897-026-00758-9 (PMC13132901; doi:10.1007/s11897-026-00758-9)
Supplement: Supplementary file 1 — (DOCX 26.6 KB) [file 11897_2026_758_MOESM1_ESM.docx]

## Supplement file 1. Search strategy

| **Search Strategy** |
| --- |

| **Databas** | **Platform** | **No of hits** | |
| --- | --- | --- | --- |
|  |  | **Search: 2025-03-31** | **Search: 2025-11-13** |
| Pubmed | Pubmed | 714 | 783 |
| EMBASE | Embase.com | 885 | 962 |
| Cinahl | EBSCOHost | 254 | 276 |
| PsycInfo | EBSCOHost | 154 | 170 |
| Scopus | Scopus | 1206 | 1327 |
| Cochrane Central | Wiley Interscience | 96 | 106 |
| **Total** |  | 4906 | 5366 |
| **Deduplicated** |  | 1597 | 1742 |

| **Search filters** | Qualitative research search filter^^[[1]](#footnote-1)^^ (modified). Systematic review search filter in PubMed^^[[2]](#footnote-2)^^ (modified) |
| --- | --- |

| **PubMed** |
| --- |

| Date of search | 2025-03-31 |
| --- | --- |
| Limits | 10 years |
| No of hits | 714 |
| *Explanation* |  |
| [MeSH] | Exploded MeSH-term |
| [tiab] | Title, Abstract (includes author keywords) |
| [pdat] | Publication date |
| “…” | Phrase search |
| * | Truncation sign (replaces any number of characters) |

(Heart-Assist Devices[MeSH]

OR

(BiVAD[tiab] OR Bi-VAD[tiab] OR HVAD[tiab] OR LVAD[tiab] OR L-VAD[tiab] OR MCS[tiab] OR VAD[tiab] OR "artificial ventricle*"[tiab] OR ”cardiac assist device*”[tiab] OR ”cardiac assist pump*”[tiab] OR "circulatory support"[tiab] OR "heart assist device*"[tiab] OR "heart assist pump*"[tiab] OR "LV assist device*"[tiab] OR ”vascular assist device*”[tiab] OR "ventric* assist device*"[tiab] OR "ventricle-assist device*"[tiab] OR "ventricular-assist device*"[tiab])

OR

("CorAide"[tiab] OR "DeBakey Child"[tiab] OR "DuraHeart"[tiab] OR "EVAHEART"[tiab] OR "FlowMaker"[tiab] OR "HeartAssist 5"[tiab] OR "HeartMate 3"[tiab] OR "HeartMate II"[tiab] OR "HeartMate III"[tiab] OR "HeartMate X"[tiab] OR "HeartMate XVE"[tiab] OR "HeartQuest"[tiab] OR "HeartWare"[tiab] OR "INCOR"[tiab] OR "Jarvik 15mm"[tiab] OR "Jarvik 2000"[tiab] OR "Levacor"[tiab] OR "Lion Heart"[tiab] OR "MiTi Heart"[tiab] OR "Novacor"[tiab] OR "Toyobo-LVASOR"[tiab] OR "Ventrassist"[tiab]))

AND

((Caregivers[MeSH] OR Family[MeSH] OR Friends[MeSH])

OR

(care giver*[tiab] OR caregiv*[tiab] OR carer*[tiab] OR caretak*[tiab] OR child[tiab] OR children[tiab] OR "close person*"[tiab] OR dyad*[tiab] OR families[tiab] OR family[tiab] OR friend*[tiab] OR husband*[tiab] OR "informal care"[tiab] OR neighbour*[tiab] OR neighbor*[tiab] OR parent*[tiab] OR partner*[tiab] OR relative[tiab] OR relatives[tiab] OR sibling*[tiab] OR "significant other*"[tiab] OR spouse*[tiab] OR wife[tiab] OR wives[tiab]))

AND

((“Focus Groups”[MeSH] OR “Interviews as Topic”[MeSH] OR Narration[MeSH] OR "Personal Narratives as Topic"[MeSH] OR Qualitative Research[MeSH])

OR

(“field research”[tiab] OR fieldwork[tiab] OR “field work”[tiab] OR “focus group”[tiab] OR “focus groups”[tiab] OR interview*[tiab] OR “key informant”[tiab] OR narration[tiab] OR narratives[tiab] OR qualitative[tiab])

OR

(Grounded Theory[MeSH] OR Hermeneutics[MeSH])

OR

(ethnograph*[tiab] OR “grounded theory”[tiab] OR hermeneutic*[tiab] OR phenomenolo*[tiab])

OR

mixed-method*[tiab] OR “multi-method stud*”[tiab] OR “multimethod stud*”[tiab]

OR

(attitude*[tiab] OR belief*[tiab] OR experience*[tiab] OR feeling*[tiab] OR perception*[tiab] OR perspective*[tiab] OR view*[tiab]))

AND

(2015:3000[pdat])

| **Embase, Embase.com** |
| --- |

| Date of search | 2025-03-31 |
| --- | --- |
| Limits | 10 years |
| No of hits | 885 |
| *Explanations* |  |
| /exp | Exploded EMTREE-term |
| :ti,ab,kw | Title, abstract, author keywords |
| :it | Terms describing publication type |
| /py | Publication year |
| '…' | Phrase search |
| * | Truncation sign(replaces any number of characters) |

('heart assist device'/exp

OR

('bivad' OR 'bi-vad' OR 'hvad' OR 'lvad' OR 'l-vad' OR 'mcs' OR 'vad' OR 'artificial ventricle*' OR 'cardiac assist device*' OR 'cardiac assist pump*' OR 'circulatory support' OR 'heart assist device*' OR 'heart assist pump*' OR 'lv assist device*' OR 'vascular assist device*' OR 'ventric* assist device*' OR 'ventricle-assist device*' OR 'ventricular-assist device*'):ti,ab,kw

OR

('coraide' OR 'debakey child' OR 'duraheart' OR 'evaheart' OR 'flowmaker' OR 'heartassist 5' OR 'heartmate 3' OR 'heartmate ii' OR 'heartmate iii' OR 'heartmate x' OR 'heartmate xve' OR 'heartquest' OR 'heartware' OR 'incor' OR 'jarvik 15mm' OR 'jarvik 2000' OR 'levacor' OR 'lion heart' OR 'miti heart' OR 'novacor' OR 'toyobo-lvasor' OR 'ventrassist'):ti,ab,kw)

AND

(('caregiver'/exp OR 'family'/exp OR 'friend'/exp)

OR

('care giver*' OR 'caregiv*' OR 'carer*' OR 'caretak*' OR 'child' OR 'children' OR 'close person*' OR 'dyad*' OR 'families' OR 'family' OR 'friend*' OR 'husband*' OR 'informal care' OR 'neighbour*' OR 'neighbor*' OR 'parent*' OR 'partner*' OR 'relative' OR 'relatives' OR 'sibling*' OR 'significant other*' OR 'spouse*' OR 'wife' OR 'wives'):ti,ab,kw)

AND

(('information processing'/de OR 'interview'/exp OR 'qualitative research'/exp OR 'verbal communication'/exp)

OR

('field research' OR 'fieldwork' OR 'field work' OR 'focus group' OR 'focus groups' OR 'interview*' OR 'key informant' OR 'narration' OR 'narratives' OR 'qualitative'):ti,ab,kw

OR

('ethnographic research'/exp OR 'grounded theory'/exp OR 'hermeneutics'/exp OR 'phenomenology'/exp)

OR

('ethnograph*' OR 'grounded theory' OR 'hermeneutic*' OR 'phenomenolo*'):ti,ab,kw

OR

('mixed-method*' OR 'multi-method stud*' OR 'multimethod stud*'):ti,ab,kw

OR

('attitude*' OR 'belief*' OR 'experience*' OR 'feeling*' OR 'perception*' OR 'perspective*' OR 'view*'):ti,ab,kw)

NOT

('conference abstract'/it OR 'conference paper'/it OR 'conference review'/it)

AND

[2015-3000]/py

| **Cinahl (EbscoHost)** |
| --- |

| Date of search | 2025-03-31 |
| --- | --- |
| Limits | 10 years |
| No of hits | 254 |
| *Explanation* |  |
| TI | Title |
| AB | Abstract |
| MH | Exact Subject Heading |
| PY | Publication year |
| '…' | Phrase search |
| * | Truncation sign (replaces any number of characters) |

((MH "Heart-Assist Devices")

OR

((TI BiVAD OR AB BiVAD) OR (TI Bi-VAD OR AB Bi-VAD) OR (TI HVAD OR AB HVAD) OR (TI LVAD OR AB LVAD) OR (TI L-VAD OR AB L-VAD) OR (TI MCS OR AB MCS) OR (TI VAD OR AB VAD) OR (TI "artificial ventricle*" OR AB "artificial ventricle*") OR (TI "cardiac assist device*" OR AB "cardiac assist device*") OR (TI "cardiac assist pump*" OR AB "cardiac assist pump*") OR (TI "circulatory support" OR AB "circulatory support") OR (TI "heart assist device*" OR AB "heart assist device*") OR (TI "heart assist pump*" OR AB "heart assist pump*") OR (TI "LV assist device*" OR AB "LV assist device*") OR (TI "vascular assist device*" OR AB "vascular assist device*") OR (TI "ventric* assist device*" OR AB "ventric* assist device*") OR (TI "ventricle-assist device*" OR AB "ventricle-assist device*") OR (TI "ventricular-assist device*" OR AB "ventricular-assist device*"))

OR

((TI CorAide OR AB CorAide) OR (TI "DeBakey Child" OR AB "DeBakey Child") OR (TI DuraHeart OR AB DuraHeart) OR (TI EVAHEART OR AB EVAHEART) OR (TI FlowMaker OR AB FlowMaker) OR (TI "HeartAssist 5" OR AB "HeartAssist 5") OR (TI "HeartMate 3" OR AB "HeartMate 3") OR (TI "HeartMate II" OR AB "HeartMate II") OR (TI "HeartMate III" OR AB "HeartMate III") OR (TI "HeartMate X" OR AB "HeartMate X") OR (TI "HeartMate XVE" OR AB "HeartMate XVE") OR (TI HeartQuest OR AB HeartQuest) OR (TI HeartWare OR AB HeartWare) OR (TI INCOR OR AB INCOR) OR (TI "Jarvik 15mm" OR AB "Jarvik 15mm") OR (TI "Jarvik 2000" OR AB "Jarvik 2000") OR (TI Levacor OR AB Levacor) OR (TI "Lion Heart" OR AB "Lion Heart") OR (TI "MiTi Heart" OR AB "MiTi Heart") OR (TI Novacor OR AB Novacor) OR (TI Toyobo-LVASOR OR AB Toyobo-LVASOR) OR (TI Ventrassist OR AB Ventrassist)))

AND

(((MH Caregivers) OR (MH Caregiving) OR (MH Family) OR (MH Siblings) OR (MH Spouses) OR (MH "Parents+"))

OR

((TI "care giver*" OR AB "care giver*") OR (TI caregiv* OR AB caregiv*) OR (TI carer* OR AB carer*) OR (TI caretak* OR AB caretak*) OR (TI child OR AB child) OR (TI children OR AB children) OR (TI "close person*" OR AB "close person*") OR (TI dyad* OR AB dyad*) OR (TI families OR AB families) OR (TI family OR AB family) OR (TI friend* OR AB friend*) OR (TI husband* OR AB husband*) OR (TI "informal care" OR AB "informal care") OR (TI neighbour* OR AB neighbour*) OR (TI neighbor* OR AB neighbor*) OR (TI parent* OR AB parent*) OR (TI partner* OR AB partner*) OR (TI relative OR AB relative) OR (TI relatives OR AB relatives) OR (TI sibling* OR AB sibling*) OR (TI "significant other*" OR AB "significant other*") OR (TI spouse* OR AB spouse*) OR (TI wife OR AB wife) OR (TI wives OR AB wives)))

AND

(((MH "Field Studies") OR (MH "Focus Groups") OR (MH "Interviews+") OR (MH Narratives+) OR (MH "Qualitative Studies+"))

OR

((TI “field research” OR AB “field research”) OR (TI fieldwork OR AB fieldwork) OR (TI "field work" OR AB "field work") OR (TI "focus group" OR AB "focus group") OR (TI "focus groups" OR AB "focus groups") OR (TI interview* OR AB interview*) OR (TI "key informant" OR AB "key informant") OR (TI narration OR AB narration) OR (TI narratives OR AB narratives) OR (TI qualitative OR AB qualitative))

OR

((MH "Ethnographic Research") OR (MH "Grounded Theory") OR (MH “Multimethod studies”) OR (MH Phenomenology) OR (MH "Phenomenological Research"))

OR

((TI ethnograph* OR AB ethnograph*) OR (TI "grounded theory" OR AB "grounded theory") OR (TI hermeneutic* OR AB hermeneutic*) OR (TI phenomenolo* OR AB phenomenolo*))

OR

((TI mixed-method* OR AB mixed-method*) OR (TI “multi-method stud*” OR AB “multi-method stud*”) OR (TI “multimethod stud*” OR AB “multimethod stud*”))

OR

(MH “Caregiver attitudes”)

OR

((TI attitude* OR AB attitude*) OR (TI belief* OR AB belief*) OR (TI experience* OR AB experience*) OR (TI feeling* OR AB feeling*) OR (TI perception* OR AB perception*) OR (TI perspective* OR AB perspective*) OR (TI view* OR AB view*)))

AND

(PY 20150101-)

| **PsycInfo (EbscoHost)** |
| --- |

| Date of search | 2025-03-31 |
| --- | --- |
| Limits | 10 years |
| No of hits | 154 |
| *Explanation* |  |
| DE | Subjects (exact) |
| TI | Title |
| AB | Abstract |
| MR | Methodology |
| PY | Publication year |
| “…” | Phrase search |
| * | Truncation sign (replaces any number of characters) |

((TI BiVAD OR AB BiVAD) OR (TI Bi-VAD OR AB Bi-VAD) OR (TI HVAD OR AB HVAD) OR (TI LVAD OR AB LVAD) OR (TI L-VAD OR AB L-VAD) OR (TI MCS OR AB MCS) OR (TI VAD OR AB VAD) OR (TI "artificial ventricle*" OR AB "artificial ventricle*") OR (TI "cardiac assist device*" OR AB "cardiac assist device*") OR (TI "cardiac assist pump*" OR AB "cardiac assist pump*") OR (TI "circulatory support" OR AB "circulatory support") OR (TI "heart assist device*" OR AB "heart assist device*") OR (TI "heart assist pump*" OR AB "heart assist pump*") OR (TI "LV assist device*" OR AB "LV assist device*") OR (TI "vascular assist device*" OR AB "vascular assist device*") OR (TI "ventric* assist device*" OR AB "ventric* assist device*") OR (TI "ventricle-assist device*" OR AB "ventricle-assist device*") OR (TI "ventricular-assist device*" OR AB "ventricular-assist device*") OR (TI CorAide OR AB CorAide) OR (TI "DeBakey Child" OR AB "DeBakey Child") OR (TI DuraHeart OR AB DuraHeart) OR (TI EVAHEART OR AB EVAHEART) OR (TI FlowMaker OR AB FlowMaker) OR (TI "HeartAssist 5" OR AB "HeartAssist 5") OR (TI "HeartMate 3" OR AB "HeartMate 3") OR (TI "HeartMate II" OR AB "HeartMate II") OR (TI "HeartMate III" OR AB "HeartMate III") OR (TI "HeartMate X" OR AB "HeartMate X") OR (TI "HeartMate XVE" OR AB "HeartMate XVE") OR (TI HeartQuest OR AB HeartQuest) OR (TI HeartWare OR AB HeartWare) OR (TI INCOR OR AB INCOR) OR (TI "Jarvik 15mm" OR AB "Jarvik 15mm") OR (TI "Jarvik 2000" OR AB "Jarvik 2000") OR (TI Levacor OR AB Levacor) OR (TI "Lion Heart" OR AB "Lion Heart") OR (TI "MiTi Heart" OR AB "MiTi Heart") OR (TI Novacor OR AB Novacor) OR (TI Toyobo-LVASOR OR AB Toyobo-LVASOR) OR (TI Ventrassist OR AB Ventrassist))

AND

((DE "Caregivers") OR (DE “Caregiving”) OR (DE "Family") OR (DE "Family Members") OR (DE "Parents") OR (DE "Partners") OR (DE "Siblings") OR (DE "Spouses") OR (DE "Husbands") OR (DE "Wives") OR (DE "Dyads") OR (DE "Significant Others") OR (TI "care giver*" OR AB "care giver*") OR (TI caregiv* OR AB caregiv*) OR (TI carer* OR AB carer*) OR (TI caretak* OR AB caretak*) OR (TI child OR AB child) OR (TI children OR AB children) OR (TI "close person*" OR AB "close person*") OR (TI dyad* OR AB dyad*) OR (TI families OR AB families) OR (TI family OR AB family) OR (TI friend* OR AB friend*) OR (TI husband* OR AB husband*) OR (TI "informal care" OR AB "informal care") OR (TI neighbour* OR AB neighbour*) OR (TI neighbor* OR AB neighbor*) OR (TI parent* OR AB parent*) OR (TI partner* OR AB partner*) OR (TI relative OR AB relative) OR (TI relatives OR AB relatives) OR (TI sibling* OR AB sibling*) OR (TI "significant other*" OR AB "significant other*") OR (TI spouse* OR AB spouse*) OR (TI wife OR AB wife) OR (TI wives OR AB wives))

AND

((DE “Content Analysis”) OR (DE "Ethnography") OR (DE "Focus Group") OR (DE "Grounded Theory") OR (DE "Hermeneutics") OR (DE "Interpretative Phenomenological Analysis") OR (DE "Narrative Analysis") OR (DE Phenomenology) OR (DE "Qualitative Methods") OR (DE "Semi-Structured Interview") OR (DE ”Thematic Analysis”)

OR

(MR ”Field Study”) OR (MR ”Focus Group”) OR (MR ”Interview”) OR (MR ”Qualitative study”)

OR

(TI ethnograph* OR AB ethnograph*) OR (TI “field research” OR AB “field research”) OR (TI fieldwork OR AB fieldwork) OR (TI "field work" OR AB "field work") OR (TI "focus group" OR AB "focus group") OR (TI "focus groups" OR AB "focus groups") OR (TI "grounded theory" OR AB "grounded theory") OR (TI hermeneutic* OR AB hermeneutic*) OR (TI interview* OR AB interview*) OR (TI "key informant" OR AB "key informant") OR (TI mixed-method* OR AB mixed-method*) OR (TI “multi-method stud*” OR AB “multi-method stud*”) OR (TI “multimethod stud*” OR AB “multimethod stud*”) OR (TI narration OR AB narration) OR (TI narrative* OR AB narrative*) OR (TI phenomenolo* OR AB phenomenolo*) OR (TI qualitative OR AB qualitative)

OR

(TI attitude* OR AB attitude*) OR (TI belief* OR AB belief*) OR (TI experience* OR AB experience*) OR (TI feeling* OR AB feeling*) OR (TI perception* OR AB perception*) OR (TI perspective* OR AB perspective*) OR (TI view* OR AB view*))

AND

(PY 20150101-)

| **Scopus** |
| --- |

| Date of search | 2025-03-31 |
| --- | --- |
| Limits | 10 years |
| No of hits | 1206 |
| *Explanation* |  |
| TITLE-ABS-KEY | Title, abstract, keywords |
| PUBYEAR | Publication year |
| “…” | Phrase search |
| * | Truncation sign (replaces any number of characters) |

(TITLE-ABS-KEY (bivad OR bi-vad OR hvad OR lvad OR l-vad OR mcs OR vad OR "artificial ventricle*" OR "cardiac assist device*" OR "cardiac assist pump*" OR "circulatory support" OR "heart assist device*" OR "heart assist pump*" OR "LV assist device*" OR "vascular assist device*" OR "ventric* assist device*" OR "ventricle-assist device*" OR "ventricular-assist device*" OR coraide OR "DeBakey Child" OR duraheart OR evaheart OR flowmaker OR "HeartAssist 5" OR "HeartMate 3" OR "HeartMate II" OR "HeartMate III" OR "HeartMate X" OR "HeartMate XVE" OR heartquest OR heartware OR incor OR "Jarvik 15mm" OR "Jarvik 2000" OR levacor OR "Lion Heart" OR "MiTi Heart" OR novacor OR toyobo-lvasor OR ventrassist))

AND

(TITLE-ABS-KEY (caregiv* OR family OR friends OR "care giver*" OR carer* OR caretak* OR child OR children OR "close person*" OR dyad* OR families OR family OR friend* OR husband* OR "informal care" OR neighbour* OR neighbor* OR parent* OR partner* OR relative OR relatives OR sibling* OR "significant other*" OR spouse* OR wife OR wives))

AND

((TITLE-ABS-KEY (ethnograph* OR "field research" OR "field work" OR fieldwork OR "focus group*” OR "grounded theor*" OR hermeneutic* OR interview* OR “key informant” OR "mixed-method*" OR “multi-method stud*” OR “multimethod stud*” OR narration OR narratives OR phenomenol* OR qualitative))

OR

(TITLE-ABS-KEY (attitude* OR belief* OR experience* OR feeling* OR perception* OR perspective* OR view*)))

AND

(PUBYEAR > 2014)

| **Cochrane** |
| --- |

| Date of search | 2025-03-31 |
| --- | --- |
| Limits | 10 years |
| No of hits | 96 |
| *Explanation* |  |
| mh | Mesh term |
| ti,ab,kw | Title, Abstract, Keyword |
| NEXT | Proximity operator (with fixed word order) |

1. [mh "Heart-Assist Devices"]

2. (BiVAD:ti,ab,kw OR Bi-VAD:ti,ab,kw OR HVAD:ti,ab,kw OR LVAD:ti,ab,kw OR L-VAD:ti,ab,kw OR MCS:ti,ab,kw OR VAD:ti,ab,kw OR ("artificial" NEXT ventricle*):ti,ab,kw OR ("cardiac assist" NEXT device*):ti,ab,kw OR ("cardiac assist" NEXT pump*):ti,ab,kw OR "circulatory support":ti,ab,kw OR ("heart assist" NEXT device*):ti,ab,kw OR ("heart assist" NEXT pump*):ti,ab,kw OR ("LV assist" NEXT device*):ti,ab,kw OR ("vascular assist" NEXT device*):ti,ab,kw OR (ventric* NEXT "assist" NEXT device*):ti,ab,kw OR ("ventricle-assist" NEXT device*):ti,ab,kw OR ("ventricular-assist" NEXT device*):ti,ab,kw )

3. (CorAide:ti,ab,kw OR "DeBakey Child":ti,ab,kw OR DuraHeart:ti,ab,kw OR EVAHEART:ti,ab,kw OR FlowMaker:ti,ab,kw OR "HeartAssist 5":ti,ab,kw OR "HeartMate 3":ti,ab,kw OR "HeartMate II":ti,ab,kw OR "HeartMate III":ti,ab,kw OR "HeartMate X":ti,ab,kw OR "HeartMate XVE":ti,ab,kw OR HeartQuest:ti,ab,kw OR HeartWare:ti,ab,kw OR INCOR:ti,ab,kw OR "Jarvik 15mm":ti,ab,kw OR "Jarvik 2000":ti,ab,kw OR Levacor:ti,ab,kw OR "Lion Heart":ti,ab,kw OR "MiTi Heart":ti,ab,kw OR Novacor:ti,ab,kw OR Toyobo-LVASOR:ti,ab,kw OR Ventrassist:ti,ab,kw)

4. #1 OR #2 OR #3

5. ([mh Caregivers] OR [mh Family] OR [mh Friends])

6. (("care" NEXT giver*):ti,ab,kw OR caregiv*:ti,ab,kw OR carer*:ti,ab,kw OR caretak*:ti,ab,kw OR child:ti,ab,kw OR children:ti,ab,kw OR ("close" NEXT person*):ti,ab,kw OR dyad*:ti,ab,kw OR families:ti,ab,kw OR family:ti,ab,kw OR friend*:ti,ab,kw OR husband*:ti,ab,kw OR "informal care":ti,ab,kw OR neighbour*:ti,ab,kw OR neighbor*:ti,ab,kw OR parent*:ti,ab,kw OR partner*:ti,ab,kw OR relative:ti,ab,kw OR relatives:ti,ab,kw OR sibling*:ti,ab,kw OR ("significant" NEXT other*):ti,ab,kw OR spouse*:ti,ab,kw OR wife:ti,ab,kw OR wives:ti,ab,kw)

7. #5 OR #6

8. ([mh "Focus Groups"] OR [mh "Interviews as Topic"] OR [mh Narration] OR [mh "Personal Narratives as Topic"] OR [mh "Qualitative Research"])

9. ("field research”:ti,ab,kw OR fieldwork:ti,ab,kw OR "field work":ti,ab,kw OR “focus group":ti,ab,kw OR "focus groups":ti,ab,kw OR interview*:ti,ab,kw OR  "key informant":ti,ab,kw OR  narration:ti,ab,kw OR narratives:ti,ab,kw OR qualitative:ti,ab,kw)

10. ([mh "Grounded Theory"] OR [mh Hermeneutics])

11. (ethnograph*:ti,ab,kw OR "grounded theory":ti,ab,kw OR hermeneutic*:ti,ab,kw OR phenomenolo*:ti,ab,kw)

12. (mixed-method*:ti,ab,kw OR (multi-metod NEXT stud*):ti,ab,kw OR (multimethod NEXT stud*):ti,ab,kw)

13. (attitude*:ti,ab,kw OR belief*:ti,ab,kw OR experience*:ti,ab,kw OR feeling*:ti,ab,kw OR perception*:ti,ab,kw OR perspective*:ti,ab,kw OR view*:ti,ab,kw)

14. #8 OR #9 OR #10 OR #11 OR #12 OR #13

15. #4 AND #7 AND #14

1. **Recherche qualitative,**<https://extranet.santecom.qc.ca/wiki/!biblio3s/doku.php?id=concepts:recherche-qualitative> (accessed 2025-03-11)  [↑](#footnote-ref-1)
2. **Search Strategy Used to Create the PubMed Systematic Reviews Filter**

   <https://www.nlm.nih.gov/bsd/pubmed_subsets/sysreviews_strategy.html> (accessed 2025-04-17)  [↑](#footnote-ref-2)
