# Supplementary material for: The Dynamic Nature of Caregiving in Advanced Heart Failure With Left Ventricular Assist Device Support: A Systematic Review and Thematic Synthesis
Source: Curr Heart Fail Rep. 2026 Apr 30;23(1):19. doi: 10.1007/s11897-026-00758-9 (PMC13132901; doi:10.1007/s11897-026-00758-9)
Supplement: Supplementary file 2 — (DOCX 17.9 KB) [file 11897_2026_758_MOESM2_ESM.docx]

## Supplement file 2. Quality appraisal of the articles included in the literature review on LVAD caregivers.

|  | Authors, Year of publishing | Clear statement of the aims of the research | Methodology appropriate | Research design appropriate to address the aims of the research | Recruitment strategy appropriate to the aims of the research? | Data collected in a way that addressed the research issue? | The relationship between researcher and participants been adequately considered | Ethical issues been taken into consideration | The data analysis was sufficiently rigorous | Clear statement of findings | Valuable |
| --- | --- | --- | --- | --- | --- | --- | --- | --- | --- | --- | --- |
| 1 | Abshire et al, 2021 | Yes | Yes | Yes | Yes | Yes | No | Yes | Yes | Yes | Yes |
| 2 | Bechtohold et al, 2024^1 JCN^ | Yes | Yes | Yes | Yes | Yes | No | Yes | Yes | Yes | Yes |
| 3 | Bechtohold et al, 2024^2 JCF^ | Yes | Yes | Yes | Yes | Yes | Yes | Yes | Yes | Yes | Yes |
| 4 | Blumenthal et al, 2015 | Yes | Yes | Yes | Yes | Yes | Yes | Yes | Yes | Yes | Yes |
| 5 | Coleman et al., 2025 | Yes | Yes | Yes | Yes | Yes | No | No | Yes | Yes | Yes |
| 6 | DeGroot et al, 2021 | Yes | Yes | Yes | Yes | Yes | No | Yes | Yes | Yes | Yes |
| 7 | Golan et al, 2023 | Yes | Yes | Yes | Yes | Yes | No | Yes | Yes | Yes | Yes |
| 8 | Keleman et al, 2024 | Yes | Yes | Yes | Yes | Yes | Yes | Yes | Yes | Yes | Yes |
| 9 | Kirkpatrick et al, 2015 | Yes | Yes | Yes | Yes | Yes | Yes | No | Yes | Yes | Yes |
| 10 | Lewis et al, 2021 | Yes | Yes | Yes | Yes | Yes | Yes | Yes | Yes | Yes | Yes |
| 11 | Magasi et al, 2019 | Yes | Yes | Yes | Yes | Yes | Yes | No | Yes | Yes | Yes |
| 12 | McIlvennan et al, 2015 | Yes | Yes | Yes | Yes | Yes | Yes | No | Yes | Yes | Yes |
| 13 | McIlvennan et al, 2016 | Yes | Yes | Yes | Yes | Yes | Yes | No | Yes | Yes | Yes |
| 14 | McIlvennan et al, 2021 | Yes | Yes | Yes | Yes | Yes | Yes | Yes | Yes | Yes | Yes |
| 15 | Neo et al, 2020 | Yes | Yes | Yes | Yes | Yes | Yes | Yes | Yes | Yes | Yes |
| 16 | Neo et al, 2021 | Yes | Yes | Yes | Yes | Yes | Yes | Yes | Yes | Yes | Yes |
| 17 | Rapelli et al, 2023 | Yes | Yes | Yes | Yes | Yes | Yes | No | Yes | Yes | Yes |
